# The Base Excision Repair Pathway Is Required for Efficient Lentivirus Integration

**DOI:** 10.1371/journal.pone.0017862

**Published:** 2011-03-23

**Authors:** Kristine E. Yoder, Amy Espeseth, Xiao-hong Wang, Qingming Fang, Maria Teresa Russo, R. Stephen Lloyd, Daria Hazuda, Robert W. Sobol, Richard Fishel

**Affiliations:** 1 Molecular Virology, Immunology, and Medical Genetics, Human Cancer Genetics, The Ohio State University Medical Center and Comprehensive Cancer Center, The Ohio State University, Columbus, Ohio, United States of America; 2 Physics Department, The Ohio State University, Columbus, Ohio, United States of America; 3 Department of Antiviral Research, Merck Research Laboratories, West Point, Pennsylvania, United States of America; 4 Department of Environment and Primary Prevention, Istituto Superiore di Sanità, Rome, Italy; 5 Center for Research on Occupational and Environmental Toxicology, Department of Molecular and Medical Genetics, Oregon Health & Science University, Portland, Oregon, United States of America; 6 Department of Pharmacology and Chemical Biology, University of Pittsburgh School of Medicine, Pittsburgh, Pennsylvania, United States of America; 7 University of Pittsburgh Cancer Institute, Hillman Cancer Center, Pittsburgh, Pennsylvania, United States of America; 8 Department of Human Genetics, University of Pittsburgh Graduate School of Public Health, Pittsburgh, Pennsylvania, United States of America; University of Minnesota, United States of America

## Abstract

An siRNA screen has identified several proteins throughout the base excision repair (BER) pathway of oxidative DNA damage as important for efficient HIV infection. The proteins identified included early repair factors such as the base damage recognition glycosylases OGG1 and MYH and the late repair factor POLß, implicating the entire BER pathway. Murine cells with deletions of the genes *Ogg1*, *Myh*, *Neil1* and *Polß* recapitulate the defect of HIV infection in the absence of BER. Defective infection in the absence of BER proteins was also seen with the lentivirus FIV, but not the gammaretrovirus MMLV. BER proteins do not affect HIV infection through its accessory genes nor the central polypurine tract. HIV reverse transcription and nuclear entry appear unaffected by the absence of BER proteins. However, HIV integration to the host chromosome is reduced in the absence of BER proteins. Pre-integration complexes from BER deficient cell lines show reduced integration activity *in vitro*. Integration activity is restored by addition of recombinant BER protein POLß. Lentiviral infection and integration efficiency appears to depend on the presence of BER proteins.

## Introduction

Retroviruses are defined by the enzymatic activities reverse transcription and integration [1]. The RNA genome of a retrovirus is reversed transcribed to a linear cDNA that is part of the poorly understood pre-integration complex (PIC). The PIC enters the nucleus where the viral enzyme integrase catalyzes the covalent attachment of the cDNA to the host genomic DNA. Lentiviruses are a subset of retroviruses that do not require cellular division for PICs to enter the nucleus, traversing the nuclear membrane by an unknown mechanism. The integration event results in the viral cDNA flanked by two single strand DNA gaps of host sequence ranging from 4–6 nucleotides and 5′ flaps of two nucleotides of viral sequence. This integration intermediate is repaired to yield the provirus flanked by 4–6 base pair duplications. It is unknown which DNA repair pathway mediates repair of the integration intermediate *in vivo*, but proteins from multiple DNA repair pathways are able to repair a similar substrate *in vitro* [2].

Several siRNA library screens have been performed to identify host factors necessary for HIV infection [3], [4], [5]. There was little overlap between the sets of host genes identified in each screen. Although each of the screens identified at least one DNA repair gene, none of these screens conclusively identified a DNA repair pathway that repairs the integration intermediate. A recent siRNA screen targeting DNA repair genes identified several proteins throughout the short patch base excision repair (BER) pathway for oxidative damage that appear to be important for HIV infection [6]. Among the proteins identified in this siRNA study were POLB, LIG3, and XRCC1, which were previously shown to efficiently repair an integration intermediate substrate *in vitro* [2].

The BER pathway begins with recognition of base damage by a DNA glycosylase, which removes the damaged base [7], [8]. Each glycosylase recognizes specific base damage; for example, Ogg1 recognizes oxidatively damaged guanine as 8-oxo-guanine (8-oxo-G) or formamidopyrimidine-guanine (Fapy-G) [8]. The abasic site left by the glycosylase is recognized by Ape1 to cleave the sugar phosphate backbone 5′ to the damaged base site. The resulting 3′ hydroxyl is extended by a polymerase, generally Polß. The remaining nick is sealed by a heterodimer of Lig3 and Xrcc1. Reduced HIV infection was associated with siRNA mediated reduced expression of the oxidative glycosylases OGG1, MYH, NTH1, NEIL2, NEIL3, the endonuclease APE1, the polymerases POLB, POLI, POLL and the ligase proteins LIG3 and XRCC1 in human cells [6].

Here we have collected mouse embryonic fibroblasts (MEFs) derived from BER gene deletion mutants, including *Ogg1*, *Myh*, *Neil1*, and *Polß* null cell lines. These cell lines and matched wild type littermate cells were infected with the gammaretrovirus Moloney MLV (MMLV) and the lentiviruses HIV and FIV. Only the lentiviruses showed reduced infection efficiency in the BER deletion cells. Quantitation of the HIV provirus shows that integration to the host genome is reduced in the absence of BER proteins. PICs derived from BER deficient cells showed reduced integration activity compared to PICs from wild type cells. PIC integration activity from Polß deficient cells was rescued with the addition of recombinant POLß protein. Oxidative damage associated BER proteins appear to affect lentiviral infection efficiency at the integration step.

## Results

### Deletion of BER genes leads to reduced lentivirus infection

Mouse strains with deletions of DNA glycosylases are viable [9], [10]. Deletion of *Polß* leads to neonatal lethality, allowing for the isolation of *Polß* null murine embryonic fibroblasts at E10–E14 [11]. It is not possible to isolate cells with deletions of the *Ape1*, *Lig3* or *Xrcc1* genes [10], [12], [13]. We have collected MEFs from mouse strains with deletions of several oxidative damage BER genes and matched wild type littermates including *Myh*, *Ogg1*, *Neil1*, and *Polß* [11], [14], [15], [16], [17]. This group of cell lines includes proteins from the initiation of BER through the late steps of BER [18].

**Figure 1:**
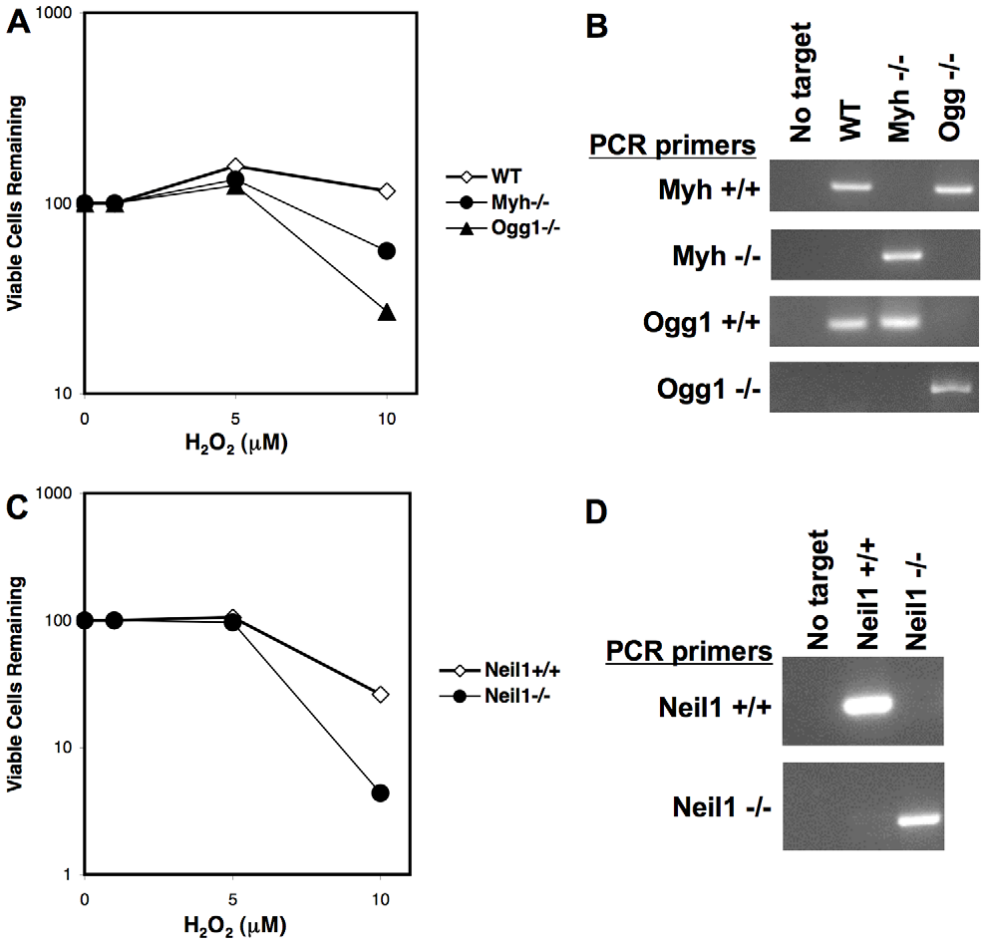
Viability of BER glycosylase deletion cell lines treated with DNA damaging agent H_2_O_2_. BER glycosylase deletion cells and matched wild type littermate cells were treated with increasing concentrations of H_2_O_2_. Cells were stained with trypan blue and viable cells counted. The percentage of viable cells remaining is shown. *(A)* Matched wild type, *Myh* null and *Ogg1* null MEFs treated with H_2_O_2_. *(B)* PCR analysis of *Myh* and *Ogg1* genotypes. Primer sets detected wild type *Myh* (Myh+/+), the *Myh* deletion construct (Myh−/−), wild type *Ogg1* (Ogg1+/+) and the *Ogg1* deletion construct (Ogg1−/−). PCR targets included water (No target) and genomic DNA from wild type (WT), *Myh*−/−, and *Ogg1*−/− cell lines. *(C)* Matched wild type and Neil1 null MEFs treated with H_2_O_2_. *(D)* PCR analysis of Neil1 genotypes. Primer sets detected wild type *Neil1* (Neil1+/+) and the *Neil1* deletion construct (Neil1−/−). PCR targets included water (No target) and genomic DNA from wild type (Neil1+/+) and *Neil1*−/− cell lines.

The DNA damage sensitivity phenotypes of the glycosylase cell lines were confirmed by treatment with varying concentrations of hydrogen peroxide (H_2_O_2_); viable cells were measured by trypan blue exclusion (Figure 1A). Myh null and Ogg1 null mice were originally crossed to generate Myh+/− Ogg1+/− mice [16], [19]. These mice were subsequently intercrossed to ultimately generate MEFs from wild type, *Myh* null, and *Ogg1* null littermates [16], [19]. Compared to matched wild type cells, *Myh* null and *Ogg1* null MEFs were sensitive to the oxidative DNA damaging agent H_2_O_2_ (Figure 1A, [17], [20]. PCR analysis of genomic DNA confirmed the genotypes of the wild type, *Myh* null, and *Ogg1* null cell lines (Figure 1B). Wild type and *Neil1* null MEFs were generated from littermates [15]. *Neil1* null MEFs were more sensitive to increasing concentrations of H_2_O_2_ compared to matched wild type MEFs (Figure 1C). The genotypes of the *Neil1* wild type and null cell lines were also confirmed by PCR (Figure 1D).

**Figure 2:**
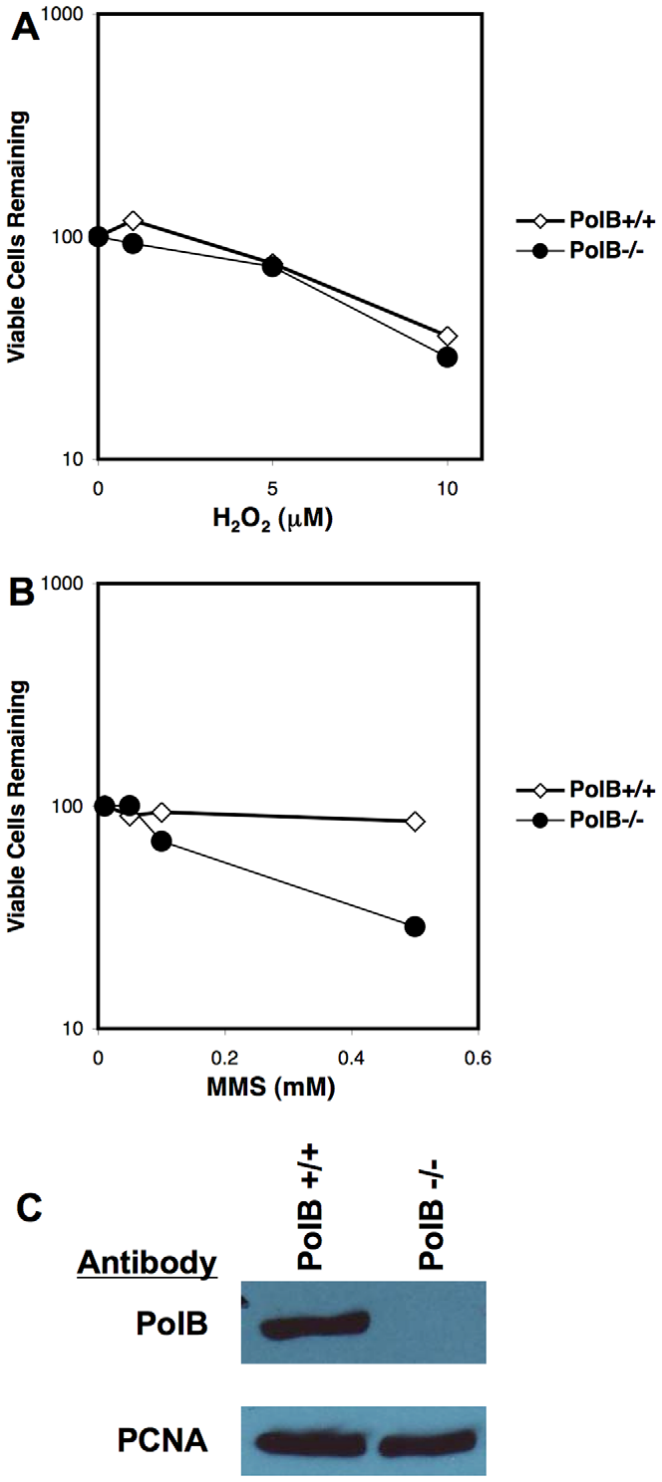
Viability of BER polymerase b cell lines treated with DNA damaging agents. *(A) Polß* deletion cells and matched wild type littermate cells were treated with increasing concentrations of the oxidative base damage inducing agent H_2_O_2_. *(B) Polß* cell lines were treated with increasing concentrations of the methylation base damage inducing agent MMS. Cells were stained with trypan blue and viable cells counted. The percentage of viable cells remaining is shown. (C) Western analysis of wild type (PolB+/+) and *Polß* null (PolB−/−) cell lines for Polß expression. Blots were stripped and re-probed for PCNA as a loading control.

The *Polß* wild type and null MEFs were derived from littermates and were tested for DNA damage sensitivity. These cells were treated with varying concentrations of H_2_O_2_ or methyl methanesulfonate (MMS); viable cells were measured by trypan blue exclusion (Figure 2, A and B). As previously reported, *Polß* null cells were sensitive to MMS, but not H_2_O_2_ [11], [21]. Immunoblot analysis confirmed expression of the Polß protein in wild type cells and absence in *Polß* null MEFs (Figure 2C). PCNA was probed as a loading control (Figure 2C).

**Figure 3:**
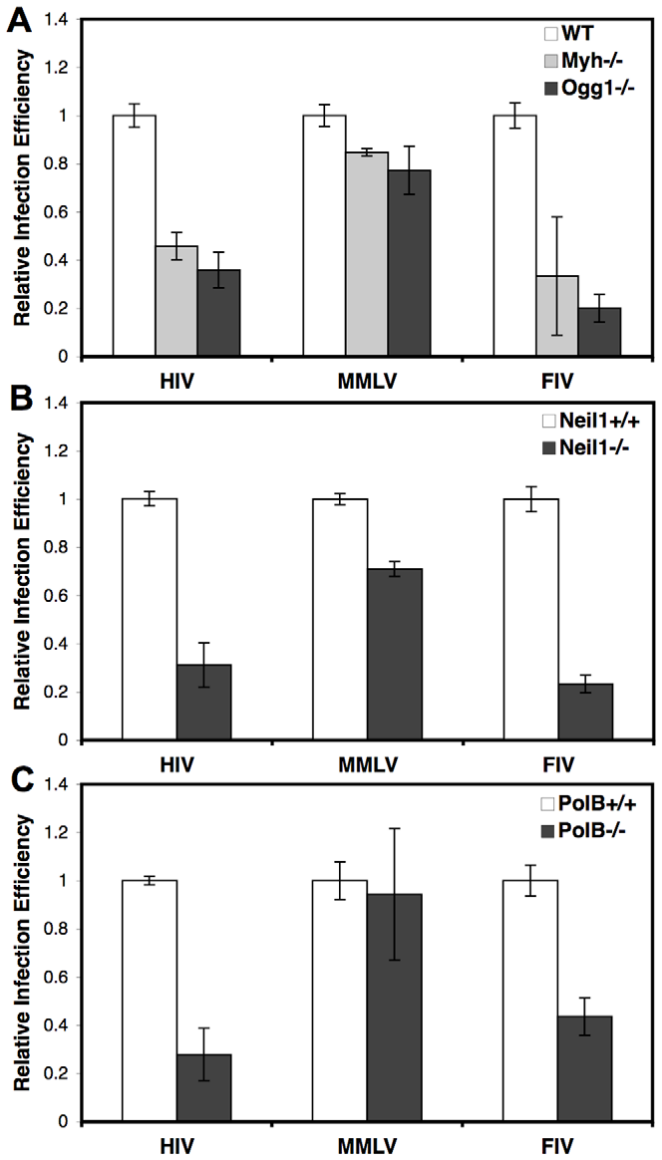
Infection of BER cell lines with HIV, MMLV, and FIV. Cells were infected with HIV, MMLV, and FIV retroviral vectors expressing GFP following integration. Cells were analyzed at 72 hpi by flow cytometry for GFP expression indicating successful infection. Wild type MEFs were from littermates. *(A)* Wild type (WT), *Myh*−/−, and *Ogg1*−/− cell lines, *(B)* Wild type (*Neil1*+/+) and *Neil1*−/− cell lines, *(C)* Wild type (*Polß*+/+) and *Polß*−/− cell lines. Infections were performed at two MOI in duplicate at least three times. Error bars indicate the standard deviation after normalization.

Wild type littermate and BER gene deletion MEFs were infected with lentiviral vectors derived from HIV and FIV and a retroviral vector derived from the gammaretrovirus MMLV (Figure 3). These vectors have been shown to faithfully recapitulate the retroviral life cycle from reverse transcription through integration [22]. The HIV, FIV, and MMLV vectors all express GFP following successful integration [23], [24], [25]. Cells were analyzed by flow cytometry for GFP expression at 72 hours post infection (hpi). Deletion of the glycosylase genes *Myh* or *Ogg1* led to decreased infection by HIV and FIV (Figure 3A; *Myh* and HIV P = 0.004, *Ogg1* and HIV P = 0.0011, *Myh* and FIV P = 0.013, *Ogg1* and FIV P = 0.0001). However, there was no significant difference between MMLV infection of wild type and *Myh* null (P = 0.072) or *Ogg1* null cells (P = 0.15). Deletion of the *Neil1* glycosylase gene also led to significant decreases in HIV (P<0.0001) and FIV (P = 0.0003) infection compared to wild type cells (Figure 3B). *Neil1* null cells did show a significant decrease in MMLV infection (P = 0.004), but the infection efficiency was less than 30% different from wild type cells (Figure 3B). Infection of cells with deletion of the DNA polymerase gene *Polß* was similar to infection of *Myh* and *Ogg1* null cells (Figure 3C, HIV P<0.0001, MMLV P = 0.31, FIV P<0.0001), suggesting that the BER pathway significantly affects lentiviral infection but not gammaretroviral infection.

### Lentiviral determinants are not associated with the BER pathway

**Figure 4:**
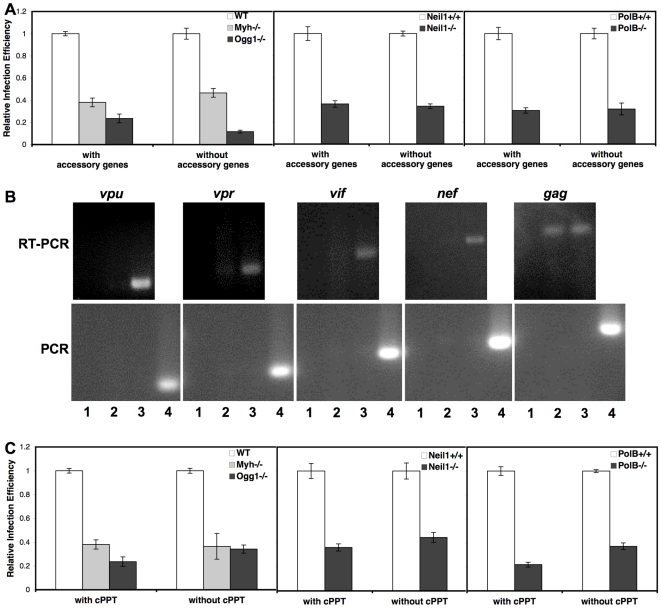
Infection of BER cell lines with HIV with and without accessory genes or the cPPT. Wild type (WT), *Myh*−/−, and *Ogg1*−/− cell lines, wild type (*Neil1*+/+) and *Neil1*−/− cell lines, or wild type (*Polß*+/+) and *Polß*−/− cell lines were infected with HIV retroviral vectors. Wild type murine embryonic fibroblasts were from littermates. Cells were analyzed at 72 hpi by flow cytometry for GFP expression. *(A)* Cells were infected with an HIV vector with accessory genes or without accessory genes (Δ*vif*Δ*vpr*Δ*vpu*Δ*nef*). *(B)* Total RNA was isolated from wild type HIV and HIV(Δ*vif*Δ*vpr*Δ*vpu*Δ*nef*) vector producer cells, treated with DNaseI to digest producer plasmids, and re-isolated. RNA fractions were amplified with the same conditions by RT-PCR or PCR, to confirm the absence of contaminating producer plasmids. RT-PCR and PCR targets were Lane 1 water negative control, Lane 2 HIV(Δ*vif*Δ*vpr*Δ*vpu*Δ*nef*) RNA, Lane 3 wild type HIV RNA, and Lane 4 wild type HIV producer plasmid DNA, positive control for PCR. Primers amplified the accessory genes *vpu*, *vpr*, *vif*, and *nef* as well as the *gag* gene. *(C)* Cells were infected with an HIV vector with or without the cPPT. Infections were performed at two MOI in duplicate at least three times. Error bars indicate the standard deviation after normalization.

The lentivirus family is a specialized subset of retroviruses [1]. One obvious difference between MMLV and HIV is the presence of accessory genes in the lentivirus, including *vif*, *vpr*, *vpu*, and *nef*. MMLV has no accessory genes. HIV vectors have been engineered to express any subset of accessory genes [26]. BER deletion cell lines were infected with HIV vector particles that did or did not include the accessory genes (Δ*vif* Δ*vpr* Δ*vpu* Δ*nef*, Figure 4A). Total RNA was purified from HIV vector particle producer cells and analyzed by RT-PCR for accessory gene expression (Figure 4B). Both wild type HIV and HIV (Δ*vif* Δ*vpr* Δ*vpu* Δ*nef*) vector producer cells express *gag* RNA, but only wild type HIV producer cells appear to produce RNA encoding the accessory genes (Figure 4B). The infection efficiency of HIV without accessory genes was similar to HIV expressing all accessory genes, indicating that the BER proteins are not interacting with lentiviral accessory genes (Figure 4A).

A second notable difference between retroviruses and lentiviruses is the presence of a central polypurine tract (cPPT) [1]. The linear cDNA of lentiviruses has a 5′ flap of single stranded DNA resulting from strand displacement synthesis during reverse transcription, which is absent in MMLV retroviral cDNA. This single stranded DNA has been shown to be a substrate for the human DNA repair enzyme FEN1 and has been suggested to mediate nuclear import of the HIV PIC [24], [27]. The wild type and BER deletion MEFs were infected with HIV vectors with and without the cPPT and analyzed for GFP expression by flow cytometry (Figure 4B). A similar pattern of infection efficiency was seen in the wild type and matched BER mutant cell lines, indicating that the BER proteins are not interacting with the lentiviral cPPT.

### BER proteins do not affect reverse transcription or nuclear import

**Figure 5:**
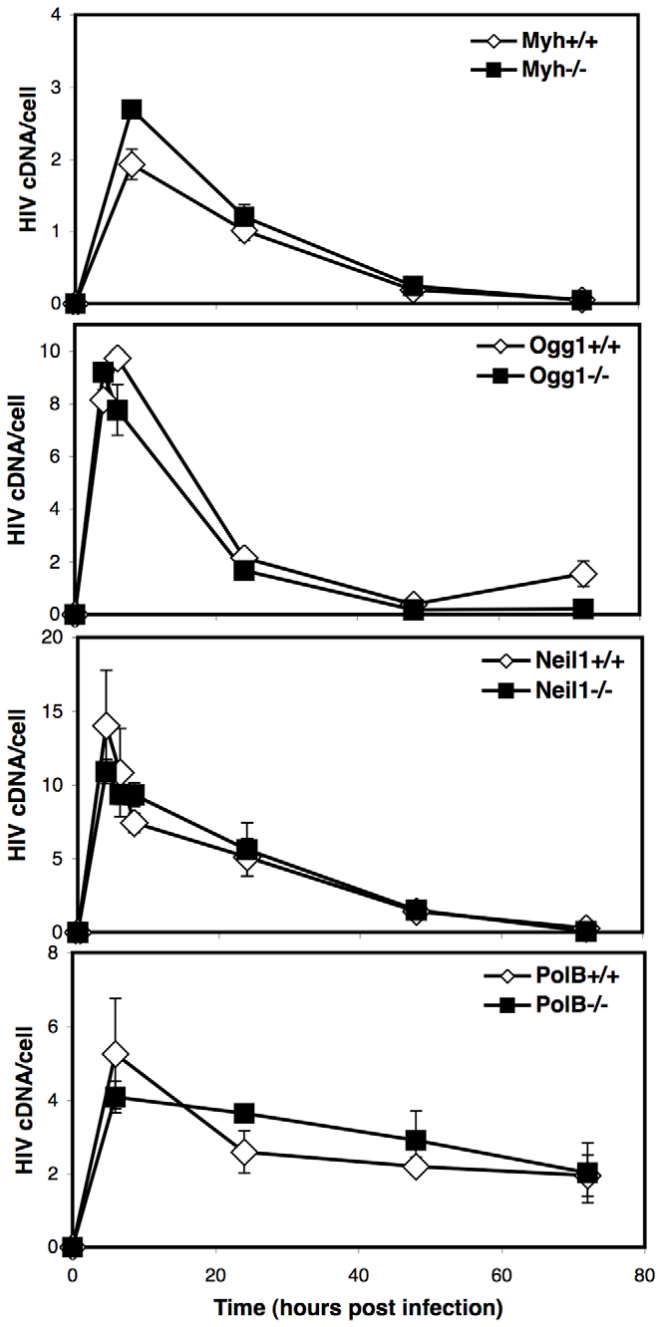
Time course of HIV cDNA accumulation in BER cells. BER wild type and deletion cell lines were infected with HIV. DNA was purified at multiple time points and analyzed by qPCR for HIV late reverse transcripts and the 18S gene. The 18S gene is a measure of the number of genomes present. The late reverse transcript primer set amplifies all complete HIV cDNAs including linear, 1LTR and 2LTR circles, and integrated provirus. The number of late reverse transcripts was divided by the number of genomes to yield HIV cDNA per cell (HIV cDNA/cell). Infections were performed in duplicate three times. Error bars indicate the standard deviation.

To determine any effect of BER proteins on reverse transcription efficiency, we evaluated the accumulation of HIV cDNA over time. The BER WT and null cell lines were infected with an HIV vector, DNA fractions were collected at multiple time points, and analyzed by quantitative PCR (qPCR) for late reverse transcripts (Figure 5). The late reverse transcript primer set spans the reverse transcription primer binding site and amplifies all cDNA forms including complete linear cDNA, 1LTR and 2LTR circles, and integrated provirus [28]. The total HIV cDNA accumulation over time was similar for *Myh*, *Ogg1*, *Neil1*, or *Polß* null and matched wild type cell lines, indicating that reverse transcription is not affected by the BER pathway.

**Figure 6:**
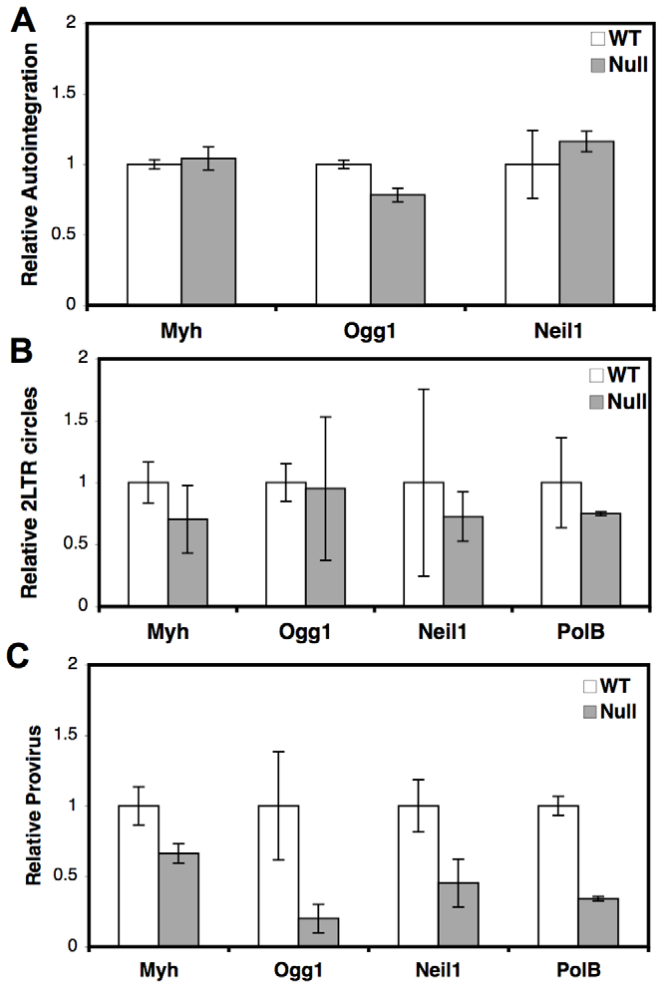
HIV autointegration, 2LTR circles, and integrated provirus in BER cells. BER wild type and deletion cell lines were infected with HIV. DNA was purified at 24 hpi or 72 hpi and analyzed by qPCR for the indicated HIV cDNA and the 18S gene. The number of HIV cDNA was divided by the number of genomes, measured by the 18S gene, to yield the HIV cDNA products per cell. Each HIV cDNA per cell is expressed relative to matched wild type cells. *(A)* HIV autointegration products at 24 hpi. *(B)* HIV 2LTR circles at 24 hpi. *(C)* HIV integrated provirus at 72 hpi. Infections were performed in duplicate three times. Error bars indicate the standard deviation after normalization.

A previous report suggested that the BER protein Ape1, as part of the cytoplasmic SET complex, protects HIV from autointegration [29]. Ape1 also plays an essential role in BER in the nucleus and a likely role in the mitochondria [8], [30]. To determine if the BER DNA glycosylases might also act by preventing HIV autointegration, DNA was analyzed by qPCR for autointegration products at 24 hpi (Figure 6A). There was no difference in HIV autointegration products between wild type and BER DNA glycosylase deletion cells.

Retroviral 2LTR circles are only found in the nuclear compartment and are an indicator of successful nuclear import of the retroviral PIC [31]. DNA from infected cells at 24 hpi was analyzed for 2LTR circles by qPCR (Figure 6B). There was no significant difference in the accumulation of 2LTR circles between wild type and BER deletion cells, indicating that the BER proteins do not affect nuclear import of HIV cDNA.

The integrated HIV provirus was also measured by qPCR in BER cell lines (Figure 6C). DNA at 72 hpi was amplified by primers to HIV and host Alu elements and further measured by qPCR [32]. While there appears to be no difference in reverse transcription, autointegration, or nuclear import of HIV cDNA, the BER mutant cell lines show reduced integrated provirus compared to wild type cells (Figure 6C). This data suggests that the BER proteins specifically affect the integration of HIV.

### BER proteins affect HIV PIC integration *in vitro*

**Figure 7:**
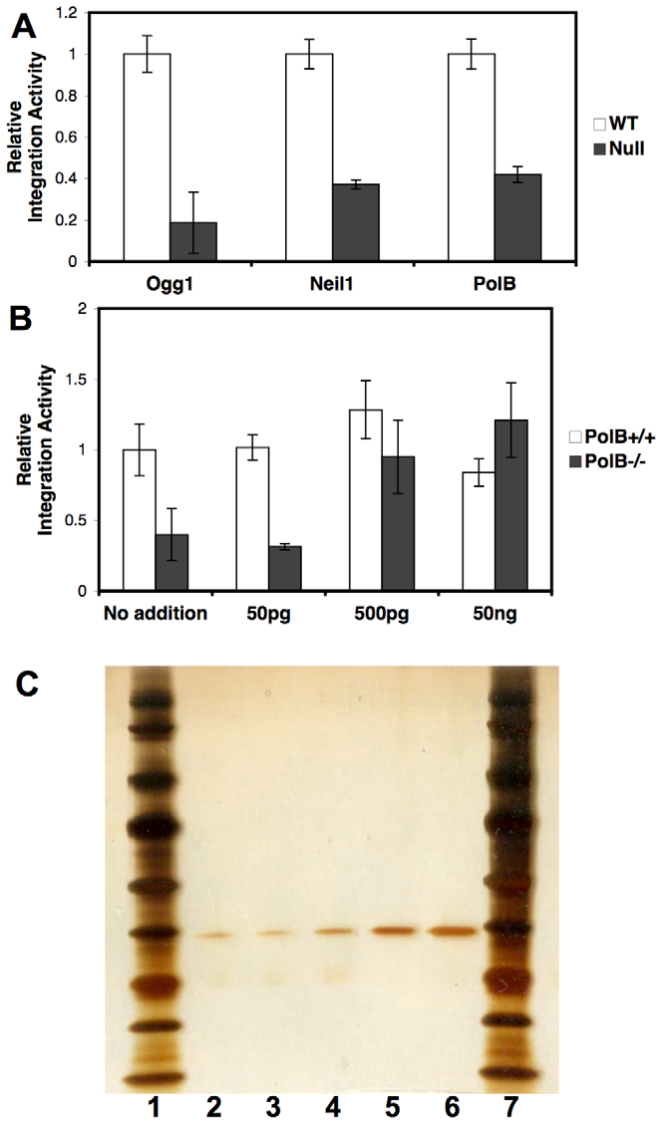
Integration activity of HIV PICs from BER cell lines. HIV PIC extracts were generated at 6 hpi. Purified human genomic DNA was added to HIV PICs. Integration products and total HIV cDNA were measured by qPCR. The number of integration products divided by the total HIV cDNA indicates the integration activity. Integration activity of PICs from mutant cells is expressed relative to PICs from matched wild type cells. *(A)* HIV PICs from matched wild type and *Ogg1*, *Neil1*, or *Polß* null cells. *(B)* Increasing concentrations of purified recombinant human POLß protein were added to PICs from wild type and *Polß*−/− cells. PICs were generated from three infections. Integration reactions for each PIC preparation were performed in duplicate. Error bars indicate the standard deviation. *(C)* Silver stained gel of recombinant human POLß protein. Lanes 1 (left) and 7 (right) show the molecular weight markers. Lanes 2–6 correspond to 20, 40, 80, 120 and 200 ng purified POLß, respectively.

The role of BER proteins during integration was further investigated with HIV PICs. HIV vector particles were added to the BER cell lines and infection was allowed to proceed for 6 hours [33]. During this time reverse transcription is completed and PICs are formed [34]. HIV PICs are fully competent to integrate into an exogenous DNA target and integration efficiency is measured by qPCR [35], [36]. HIV PICs derived from matched wild type cells were compared to PICs from *Ogg1*, *Neil1*, and *Polß* null cells (Figure 7A). Integration to purified genomic DNA was significantly reduced when either a DNA glycosylase or Polß was absent. Increasing concentrations of recombinant Polß protein was added to integration reactions of the wild type and Polß null PIC extracts (Figure 7C) [11]. The integration efficiency of HIV PICs from Polß null cells was rescued by the addition of recombinant protein (Figure 7B). The role of BER proteins during lentiviral infection appears to be associated with the integration reaction.

## Discussion

During the process of retroviral integration, the viral cDNA is covalently joined to the host chromosome but is flanked by 4–6 base pair gaps of host DNA and a 5′ dinucleotide flap of viral DNA [1]. The identities of host or viral factors that mediate repair of this integration intermediate are unknown. Three siRNA library screens of host factors that affect HIV infection efficiency failed to conclusively identify DNA repair pathways that might complete repair of the integration intermediate [3], [4], [5]. Studies of repair with recombinant proteins *in vitro* indicated that any polymerase, endonuclease and ligase could repair the integration intermediate, suggesting that multiple DNA repair pathways may mediate this process *in vivo* [2].

A recent study described an siRNA screen targeted to host DNA repair proteins [6]. This study identified multiple host genes throughout the oxidative BER pathway that were required for efficient HIV infection. Using a panel of deletion cell lines, we have found that several BER proteins affect lentiviral infection but not infection by a gamma retrovirus. The role of the BER pathway appears to be at the integration step of the viral life cycle. One obvious mechanism for BER proteins during lentiviral integration is that these proteins complete repair of the integration intermediate. It is possible that lentiviruses rely largely on BER while retroviruses are less restricted. It is not yet clear how glycosylases might be involved in repair of gapped DNA. It is possible that glycosylases target downstream BER proteins to the integration intermediate.

Other host factors have been identified that play a role during lentiviral but not retroviral infection. Significantly, LEDGF has been shown to enhance lentiviral integration by directly binding to lentiviral integrase and chromatin [37]. Mouse cells with a deletion of the *Ledgf* (*Psip1*) gene have been engineered and show a pronounced defect in lentiviral infection and no effect on retroviral infection [36]. While LEDGF is known to affect HIV integration to chromatin DNA targets, HIV PICs generated in *Ledgf* null cells have no integration defect with a naked DNA target [36]. Results with HIV PICs from BER deficient cells indicate that BER affects integration to naked DNA. The ability of BER to direct integration to chromatin targets remains to be tested. BAF and HMGA1 proteins were also shown to stimulate HIV PIC integration activity, but reduced expression of these genes showed no effect on HIV infection efficiency [38], [39], [40], [41]. This is the first example of putative HIV integration co-factors that show a difference in the integration efficiency of PICs *in vitro* and infection efficiency *in vivo*.

Retroviral integration sites display a subtle sequence preference unique to each virus [42], [43]. The HIV integration site favors G at nucleotides immediately adjacent to the attachment sites. The oxidative DNA glycosylases, with the exception of NTH1, all recognize some form of damaged G [8], [30]. Among the most common oxidative base lesions are 8-oxo-G and Fapy-G [7]. It is intriguing that the BER pathway responsible for repair of oxidative damage, largely damaged Gs, appears to be important for HIV integration and that this integration occurs preferentially at Gs. In contrast, BER apparently does not affect MMLV integration and MMLV has no preference for G/C base pairs at integration sites [42], [43]. Whether BER proteins affect the integration sites of lentiviruses is under investigation.

## Materials and Methods

### Cell lines

Murine embryonic fibroblasts used in this study have been previously described [11], [14], [15], [16], [17]. Briefly, *Ogg1* null mice were originally created in 1999 [14]. *Myh* deletion mice were later engineered in 2004 [16]. Ogg1 null and Myh null mice were crossed to generate *Myh+/− Ogg1+/−* mice. These mice were subsequently intercrossed generating offspring in predicted Mendelian ratios [19]. The wild type, *Myh* null, and *Ogg1* null MEFs were derived from littermates and spontaneously transformed [17], [19]. The wild type *Myh* gene was amplified with previously described primers P1 and P2 generating a 0.26 kb product (Table 1, [16]. Deletion of the *Myh* gene was amplified with primers P1 and *Neo* gene primer P3 generating a 0.38 kb product (Table 1, [16]. The wild type *Ogg1* gene was amplified with previously described primers Ogg1-2 and Ogg1-12 yielding a 0.14 kb product (Table 1, [16]. The deletion of *Ogg1* was confirmed with PCR primers Ogg1-3 and *Neo* gene primer N10 yielding a 1.1 kb product (Table 1, [16].

**Table 1:** Primers used in this study.

Target	Primer name	Primer sequence	Reference
*Myh*	P1	CAAGTGCTGGGATCAAAGGTG	[16]
*Myh* wild type	P2	GCTCCTTCTTGTAGCCGACG	[16]
*Myh* null	P3	TCCTCGTGCTTTACGGTATCG	[16]
*Ogg1* wild type	Ogg1-2	GCCTTGTGGGCCTCTTCATA	[16]
*Ogg1* wild type	Ogg1-12	CACCTGAGGAAGTTGGGCC	[16]
*Ogg1* null	Ogg1-3	CAAGACCCCACTGAGTGCC	[16]
*Ogg1* null	N10	GAGAACCTGCGTGCAATCCA	[16]
*Neil1*	Neil forward	CACCAGTGAGCAAGACAGCCAT	[15]
*Neil1* wild type	RP-WT	GTGGCTGGCCAGGTGCAGCTC	[15]
*Neil1* null	RP-KO	GGGCTGACCGCTTCCTCGTGC	[15]
*vpu*	KY586	GCAATAGTTGTGTGGTCCATAG	this work
*vpu*	KY587	CAACATCCCAAGGAGCATGG	this work
*vpr*	KY588	GAGGAGCTTAAGAATGAAGCTG	this work
*vpr*	KY589	CTACTGGCTCCATTTCTTGC	this work
*vif*	KY590	CATACAGGAGAAAGAGACTGGC	this work
*vif*	KY591	TAACACTAGGCAAAGGTGGC	this work
*nef*	KY592	CAAGGCAGCTGTAGATCTTAGC	this work
*nef*	KY593	CGGCTGTCAAACCTCCACTC	this work
*gag*	KY594	ACCCGGCCATAAAGCAAGAG	this work
*gag*	KY595	CCTTGTCTATCGGCTCCTGC	this work

The mouse *Neil1* gene was deleted and crosses of heterozygous mice generated offspring in Mendelian ratios of 1∶2∶1 [15]. Wild type and *Neil1* null MEFs were generated from littermates and immortalized with the adenovirus *E1A* gene [44]. The wild type *Neil1* gene was PCR amplified with previously described primers Neil1 forward and reverse RP-WT yielding a 265 bp product (Table 1, [15]. Deletion of the *Neil1* gene was PCR amplified with the forward primer and reverse RP-KO generating an 804 bp product (Table 1, [15].

Wild type and *Polß* null MEFs were derived from littermates and immortalized with SV40 large T antigen [11], [45]. The *Polß* null cells are wild type for DNA polymerase iota [45], [46].

All cell lines were cultured at 37°C in a humidified incubator with 10% CO_2_ in DMEM supplemented with 10% fetal bovine serum, penicillin, streptomycin, and glutamax. All media reagents were from Invitrogen.

### Cell extract preparation and immunoblot assays

Nuclear extracts were prepared using the NucBuster nuclear protein extraction reagent (Novagen). Protein concentration was determined using Bio-Rad protein assay reagents according to the manufacturer's instructions. Nuclear protein (30 µg) was separated by 4–12% SDS-PAGE and electrotransferred to a 0.45 mm nitrocellulose membrane (Trans-Blot, Bio-Rad). Antigens were detected using standard protocols. Primary antibodies anti-Polß (NeoMarkers, #MS-1402-P0) and anti-PCNA (Santa Crus #sc-56) were diluted 1000×. The HRP conjugated secondary antibody (GAM-HRP or GAR-HRP, Bio-Rad) was diluted 10,000× in TBST/5% milk. Each membrane was stripped and re-probed with anti-PCNA antibodies to correct for differences in protein loading.

### Retroviral vectors

Retroviral vectors were generated by transfecting 293T cells (ATCC) with three plasmids: a VSV-G envelope protein plasmid, a packaging construct expressing retroviral structural and enzymatic genes, and a genomic RNA plasmid [47]. Media containing the retroviral vector particles was collected, filtered to remove producer cells, and treated with DNaseI to digest producer plasmids.

The HIV packaging construct has a deletion in the *env* gene but expresses all accessory genes [26]. The HIV Δ*vif* Δ*vpr* Δ*vpu* Δ*nef* packaging construct has deletions of the accessory genes but expresses *tat* and *rev* genes [26]. Both packaging constructs were sequenced to confirm the absence of accessory genes in Δ*vif* Δ*vpr* Δ*vpu* Δ*nef* and the presence of complete open reading frames of *vif*, *vpr*, *vpu*, and *nef*. The HIV genomic RNA plasmid p156RRLsinPPTCMVGFPPRE includes the cPPT [24]. The cPPT was removed by digesting with ClaI and HpaI, filling in with Klenow, and ligating. Both genomic RNA plasmids were sequenced to confirm the presence or absence of the cPPT. The FIV packaging (pFP93) and genomic RNA (pGiNSiN) plasmids have been described [25]. The MMLV retroviral vectors were generated with pHIT60 and pLEGFP-C1 [23] and Clontech).

To determine infection efficiency cells were plated in 6 well dishes to achieve equivalent cell densities and verified by counting. Cells were infected in duplicate with the retroviral vectors at two MOI in the presence of 10 µg/ml DEAE dextran (Sigma Aldrich) and the media was replaced at 2 hpi. The infected cells were incubated for 72 hours then trypsinized, fixed with paraformaldehyde (Sigma Aldrich), and analyzed for GFP expression by flow cytometry (BD FACS Calibur and CellQuest software). The percentage of GFP positive mutant cells was normalized to wild type cells to obtain the relative infection efficiency. Flow cytometry data from figure 2 was analyzed by paired *t* test to generate two-tail P values (GraphPad Prism 4, San Diego). P values were rounded to two significant figures.

To confirm the expression of HIV accessory genes, RNA was isolated from 293T producer cells following transfection (Qiagen RNeasy kit). RNA samples were treated with DNaseI to digest any producer plasmids (Roche). RNA was re-isolated (Qiagen RNeasy kit) and amplified by RT-PCR (Superscript One-Step RT-PCR with Platinum Taq, Invitrogen) or PCR (Platinum Taq, Invitrogen). PCR with Platinum Taq was performed to show that RNA samples did not contain DNA from producer plasmids, a positive control reaction with the HIV packaging construct was included. 25 µl RT-PCR and PCR reactions utilized primers listed in Table 1 with the predicted product sizes of 185 bp for *vpu*, 220 bp for *vpr*, 285 bp for *vif*, 320 bp for *nef*, and 380 bp for *gag*. Reaction products were analyzed by agarose gel stained with ethidium bromide.

### Quantitative PCR

At indicated times following infection with HIV, cells were trypsinized and DNA was purified (Qiagen DNeasy Blood and Tissue Kit). The HIV late reverse transcript, 2LTR circle, and provirus primer sets have been described [28], . The nested PCR method for detection of HIV autointegration products has been described [29], but primer sets were modified for amplification of the HIV retroviral vector. The first PCR reaction included primers MH532, KY214 5′ CCATCTTCTTCAAGGACGAC 3′, and KY215 5′ GTCGTCCTTGAAGAAGATGG 3′ and the second PCR amplification included primers MH535, SB-76, and probe MH603 [28], [48]. The number of cell genomes was determined by qPCR of the 18S gene (Applied Biosystems). The number of HIV cDNA products was divided by the number of cell genomes to yield the number of HIV cDNA forms per cell. Reactions were performed in triplicate. Standards for absolute quantitation were known amounts of plasmid standards or cellular genomes. QPCR was performed with Taqman mastermix in an Applied Biosystems 7900HT Sequence Detection System.

### Pre-integration complexes

HIV PICs were generated as previously described [33]. Briefly, BER cell lines were infected with HIV vector particles and incubated for 6 hours. Cells were trypsinized, washed with Buffer K (20 mM HEPES, pH 7.4, 150 mM KCl, 5 mM MgCl_2_), and lysed in Buffer K with 0.5% NP-40, 1 mM DTT, and protease inhibitors. Extracts were spun at 3000×*g* and 10,000×*g*, supernatants were frozen in liquid nitrogen and stored at −80°C. Integration reactions included PIC extract and 100 ng human genomic DNA at 37°C for 1 hour. DNA was purified following integration (Qiagen DNeasy Blood and Tissue Kit). Recombinant human Polß was purified as described [49].

### Recombinant human Polß protein

Highly active human Polß protein was kindly provided to RWS by S.H. Wilson (NIEHS) and was purified as described [50]. The purity of Polß was determined by SDS-PAGE followed by silver staining. Different amounts of purified Polß was loaded onto 4–12% precast NuPAGE Tris-glycine gels (2 hrs @ 125V) and stained with a SilverSNAP Stain Kit II (Thermo Scientific, Rockford, IL).
